# COVID-19 disease severity to predict persistent symptoms: a systematic review and meta-analysis

**DOI:** 10.1017/S1463423622000585

**Published:** 2022-11-10

**Authors:** Emre Dirican, Tayibe Bal

**Affiliations:** 1 Faculty of Medicine, Department of Biostatistics, Hatay Mustafa Kemal University, Hatay, Turkey; 2 Faculty of Medicine, Department of Infectious Diseases and Clinical Microbiology, Hatay Mustafa Kemal University, Hatay, Turkey

**Keywords:** COVID-19, disease severity, persistent, symptoms

## Abstract

**Background:**

It is unclear, whether the initial disease severity may help to predict which COVID-19 patients at risk of developing persistent symptoms.

**Aim:**

The aim of this study was to examine whether the initial disease severity affects the risk of persistent symptoms in post-acute COVID-19 syndrome and long COVID.

**Methods:**

A systematic search was conducted using PUBMED, Google Scholar, EMBASE, and ProQuest databases to identify eligible articles published after January 2020 up to and including 30 August 2021. Pooled odds ratio (OR) and confidence intervals (CIs) were calculated using random effects meta-analysis.

**Findings:**

After searching a total of 7733 articles, 20 relevant observational studies with a total of 7840 patients were selected for meta-analysis. The pooled OR for persistent dyspnea in COVID-19 survivors with a severe versus nonsevere initial disease was 2.17 [95%CI 1.62 to 2.90], and it was 1.33 [95%CI 0.75 to 2.33] for persistent cough, 1.30 [95%CI 1.06 to 1.58] for persistent fatigue, 1.02 [95%CI 0.73 to 1.40] for persistent anosmia, 1.22 [95%CI 0.69 to 2.16] for persistent chest pain, and 1.30 [95%CI 0.93 to 1.81] for persistent palpitation.

**Conclusions:**

Contrary to expectations, we did not observe an association between the initial COVID-19 disease severity and common persistent symptoms except for dyspnea and fatigue. In addition, it was found that being in the acute or prolonged post-COVID phase did not affect the risk of symptoms. Primary care providers should be alert to potential most prevalent persistent symptoms in all COVID-19 survivors, which are not limited to patients with critical–severe initial disease.

## Introduction

Coronavirus disease 2019 (COVID-2019) is caused by the severe acute respiratory syndrome coronavirus 2 (SARS-CoV-2), a novel betacoronavirus of the genus *Coronavirus*, the family *Coronaviridea*, and subfamily *Orthocoronavirinea* (Kim *et al.*, 2021). According to the World Health Organization (WHO), there were more than 225 million confirmed COVID-19 cases including more than 4.6 million deaths globally as of 15 September 2021 (WHO, 2021). The clinical impact of COVID-19 infection ranges from mild disease to life-threatening ARDS (WHO, 2020).

Recent advances in the field of COVID-19 disease such as the widespread implementation of COVID-19 vaccines have led to an increase in the number of patients recovering from COVID-19 (Rossman *et al.*, 2021). However, a substantial proportion of these will experience persistent and prolonged residual effects of COVID-19 disease after recovery from acute infection (Cabrera Martimbianco *et al.*, 2021). As a result, a large body of literature has emerged around the problem of finding optimal strategies for this population, which is a new challenge for researchers.

There is increasing clarity in the literature on the prevalence of post-acute and long COVID symptoms in COVID-19 survivors (Cabrera Martimbianco *et al.*, 2021; Sanchez-Ramirez *et al.*, 2021). Nevertheless, there is less/no clarity on the factors that determine this risk, such as the initial disease severity (Huang *et al.*, 2021b; Townsend *et al.*, 2021). Answers to these question may help to guide healthcare professionals in improving early symptom recognition of post-acute and long COVID symptoms, timely intervention and provide a general consensus to avoid wasting scarce resources in health and social care.

Although there are several studies in the literature comparing the risks of the most prevalent persistent symptoms (dyspnea, fatigue, cough, chest pain, anosmia and palpitation) of post-acute and long COVID between the different disease severity levels, the results are inconsistent (Halpin *et al.*, 2021; Sanchez-Ramirez *et al.*, 2021). Therefore, the aim of the study was to estimate symptom-specific pooled associations of the initial disease severity with the risk of persistent symptoms in post-acute and/or long COVID by conducting a meta-analysis of the available literature.

## Materials & methods

### Protocol and registration

This systematic review was performed according to the Preferred Reporting Items for Systematic Reviews and Meta-analysis (PRISMA) statement (Moher *et al.*, 2009). The systematic review protocol was registered with the International Prospective Register of Systematic Reviews (PROSPERO; registration number CRD42021272990).

### Eligibility criteria

Studies were included if they were peer-reviewed, observational (cohort and cross-sectional) studies written in English, included adult (age ≥ 18 years) COVID-19 patients (confirmed by nasopharyngeal swab or sputum RT-PCR), compared the prevalence of persistent symptoms between patient recovery after COVID-19 with severe and nonsevere initial disease in post-acute and/or long COVID, and provided the population statistics, which would allow calculation of the odds ratio (OR) of persistent symptoms according to disease severity.

Studies not published in a peer-reviewed journal, with no laboratory confirmation evidence of SARS-CoV-2 infection, with no comparison group regarding disease severity, with a follow-up time shorter than four weeks after symptom onset/admission or three weeks after discharge/recovery, including patients younger than 18 years, case reports, and those which did not include original data (meta-analysis, reviews, editorials, and opinions) were excluded.

### Definitions used

The initial disease severity was defined according to WHO, Clinical Management of COVID-19: Interim Guidance (WHO, 2020). COVID-19 survivors with a mild/moderate initial disease were classified as nonsevere, while patients with a severe/critical initial disease were classified as severe initial disease. As these definitions differ between the included studies, how we decided on binary classification (severe and nonsevere) is detailed in Table 1. The definition of post-acute COVID-19 syndrome include any COVID-19 survivors who still had signs and symptoms of COVID-19 at 4 to 12 weeks after acute infection. Long COVID included any COVID-19 survivors with symptoms persisting beyond 12 weeks of the onset of acute infection (Nalbandian *et al.*, 2021).

**Table 1.** Main characteristics of the studies

Study	Country	Setting	Sample Size	**Age** ^*^	Females	ICU Admission	Obesity (BMI ≥ 30)	CS use	Follow-up	Initial disease severity (severe versus nonsevere)	Quality score
Garrigues *et al.*, 2020	France	Single center	120	63.2	45 (37.5)	24 (20.0)	NR	NR	110 days, PA	ICU versus ward	5
De Graaf *et al.,* 2021	Netherlands	Single-center	81	60.8	30 (37)	34 (42)	NR	3 (0.4)	6 weeks, PD	ICU versus non-ICU	5
Halpin *et al.,* 2021	UK	Single-center	100	66.6	46 (46.0)	32 (32.0)	24 (24.0)	NR	4–8 weeks, PD	ICU versus. ward	5
Huang *et al.*, 2021a	China	Single-center	1733	57	836 (48)	76 (4)	NR	398 (23)	6 months, PSO	^**^Scale 3–4 versus Scale 5–6	6
Huang *et al.*, 2021b	China	Cohort, single-center	1276	59	595 (47)	54 (4)	NR	307 (24)	12 months, PSO	^**^Scale 3–4 versus Scale 5–6	6
Iqbal *et al.,* 2021	Pakistan	Single-center	158	40.1	87 (55.1)	13 (8.2)	NR	NR	38.1 days, PR	Severe versus mild/moderate	7
Jacobson *et al.,* 2021	USA	Single-center	118	43.3	55 (46.6)	11 (9.3)	NR	0 (0)	3–4 months, PD	Hospitalized versus non-hospitalized	4
Lerum *et al.,* 2021	Norway	Prospective, multicenter	103	59	49 (47.5)	15 (15)	NR	NR	3 months, PA	ICU versus non-ICU	6
Lombardo *et al.,* 2021	Italy	Cohort, multicenter	303	53	165 (54)	8 (2.6)	49 (16)	14 (4.6)	12 months, PR	Hospitalized versus non-hospitalized	5
Lorenzo *et al.,* 2020	Italy	Prospective and retrospective single center	185	57	62 (33.5)	4 (2.2)	NR	NR	23 days, PD	Hospitalized versus discharged from ED	6
Maestre-Muñiz *et al.,* 2021	Spain	Single-center	766	65.1	378 (49.3)	NR	139 (18.1)	NR	1 year, PD	Hospitalized versus non-hospitalized	6
Meije *et al.,* 2021 a,b	Spain	Prospective, single-center	294/302	68.8	131 (43.4)	27 (8.9)	41 (13.6)	125 (41.4)	45 days/7 months, PD	PaO2/FiO2 > 300 versus PaO2/FiO2 ≤ 300	5
Menges *et al.,* 2021	Switzerland	Prospective longitudinal cohort, single-center	431	47	214 (49.7)	10 (2.3)	NR	NR	220 days, PD	Hospitalized versus non-hospitalized	4
Morin *et al.,* 2021	France	Prospective, single-center	478	61	201 (42.1)	142 (29.7)	130/351 (37.0)	24 (5.0)	4 months, PD	Intubated versus non-intubated	7
Qin *et al.,* 2021	China, France, Russia	Prospective, multi-center	647	58	362 (56)	NR	NR	NR	3 months, PD	Severe versus nonsevere	6
Shendy *et al.,* 2021	Egypt	Single-center	81	34	55 (67.9)	NR	35 (43.2)	NR	3–5 months PR	Hospitalized versus non-hospitalized	7
Sykes *et al.,* 2021	UK	Single-center	134	59.6	46 (34.3)	27 (20)	NR	NR	113 days, PD	ICU versus ward	5
Voruz *et al.,* 2021	Swittzerland	Prospective, single-center	45	56.9	19 (42.2)	15 (33.3)	NR	NR	6–9 months, PSO	Severe versus mild/moderate	6
Wang *et al.,* 2020	China	Prospective, single-center	131	49	72 (54.9)	NR	NR	NR	4 weeks, PD	Severe versus nonsevere	4
Zayet *et al.,* 2021	France	Retrospective observational cohort, single-center	354	49.6	223 (63)	5 (11.1)	NR	NR	9 months, PSO	Hospitalized versus non-hospitalized	5

ED = emergency department, PA = post-admission; PSO = post-symptom onset; PD = post-discharge; PR = post-recovery, NR = not reported, CS = corticosteroid, PaO2 = partial pressure arterial oxygen, FiO2 = fraction of inspired oxygen.

* Median or mean ages (years).

** **Scale 3**: not requiring supplemental oxygen; **Scale 4:** requiring supplemental oxygen; or **Scale 5–6**: requiring high-flow nasal cannula, noninvasive mechanical ventilation, or invasive mechanical ventilation.

### Databases and search strategy

A systematic search was conducted using PUBMED, Google Scholar, EMBASE, and ProQuest databases to identify eligible articles published after January 2020 up to and including August 302 021. The search strategy was formulated using the terms: ‘dyspnea’, ‘cough’, ‘fatigue’, ‘anosmia’, ‘chest pain’, ‘palpitation’ in combination with ‘long’ or ‘persistent’ or ‘chronic’ or ‘prolonged’ or ‘survivor’ or ‘post-acute’ or ‘post-recovery’ or ‘post-discharge’ and ‘COVID’ (including ‘COVID-19’, ‘coronavirus disease 2019’, ‘2019-nCoV’, ‘SARS COV2’, ‘Covid 19’) not ‘children’.

### Study selection and data extraction

Titles and abstracts were first screened independently by ED and TB based on the inclusion and exclusion criteria with any disagreement resolved by consensus. Next, the full text of the selected studies was independently screened by two authors (ED and TB) for inclusion eligibility. The results were checked and discussed by ED and TB to agree on the final decision, and any disagreement was again resolved by consensus.

From the included studies, data were extracted on the study characteristics (author, year, country of origin, study design, sample size, type [‘post-admission’ or ‘post-discharge’ or ‘post-recovery’ or ‘post-symptom onset’], and length of follow-up), patient characteristics (mean or median age, gender, definition of acute COVID-19 infection, history of corticosteroid use, obesity status, and intensive care unit requirement), and patient clinical features (dyspnea, cough, fatigue, anosmia, chest pain, and palpitation). All data and details on binary categories (severe versus nonsevere initial disease) were collected using specific data collection forms by two authors (ED and TB) independently, in duplicate with any disagreements resolved through discussion. Missing data were excluded from the analysis of that particular variable.

### Bias risk assessment

Minimizing the potential for bias in a meta-analysis, such as sampling or measurement bias, can help avoid underestimating or overestimating the parameter of interest (Hoy *et al.*, 2012). In this study, the quality assessment was made with the Newcastle–Ottawa Scale (NOS) (GA Wells *et al.*, 2021). Studies were scored on overall quality ranging from 0 (minimum) to 9 (maximum) stars according to the assessment of the NOS scale criteria. Studies with a quality score ≤4 were categorized as low-quality studies, and those with a score of ≥5 were categorized as high-quality studies (Xie *et al.*, 2021). Publication bias was evaluated by visual inspection of the asymmetry of the funnel plots and analyzed with Egger’s linear regression, and a *P*-value of <0.05 was considered statistically significant publication bias.

### Statistical approach

In this study, the pooled effect (OR) refers to the risk of persistent symptoms in COVID-19 survivors with a severe compared to nonsevere initial disesase. The pooled OR and confidence intervals (CI) were calculated based on the Mantel–Haenszel (MH) and inverse variance (I) methods. Final results were given with the MH method as there were very minor differences between the MH and I methods for the present study.

Meta-analysis was conducted using the R v3.6.1 packages ‘meta v4.10-0’, ‘metafor v2.1-0’, and ‘PRISMAstatement v1.1.1’. All meta-analyses were performed using the random effects model. Meta-analysis was performed separately for each variable. To determine the heterogeneity between studies, *I* ^2^ statistic (*I* ^2^ >%75 significant heterogeneity), Cochran’s Q (Q) value and significance (significant *P* value indicates heterogeneity) were used. Meta-regression was performed to explore the potential variable (12-week period), contributing to heterogeneity. Forest plots were used to illustrate results from relevant articles.

## Results

### Study selection

**Figure 1.**
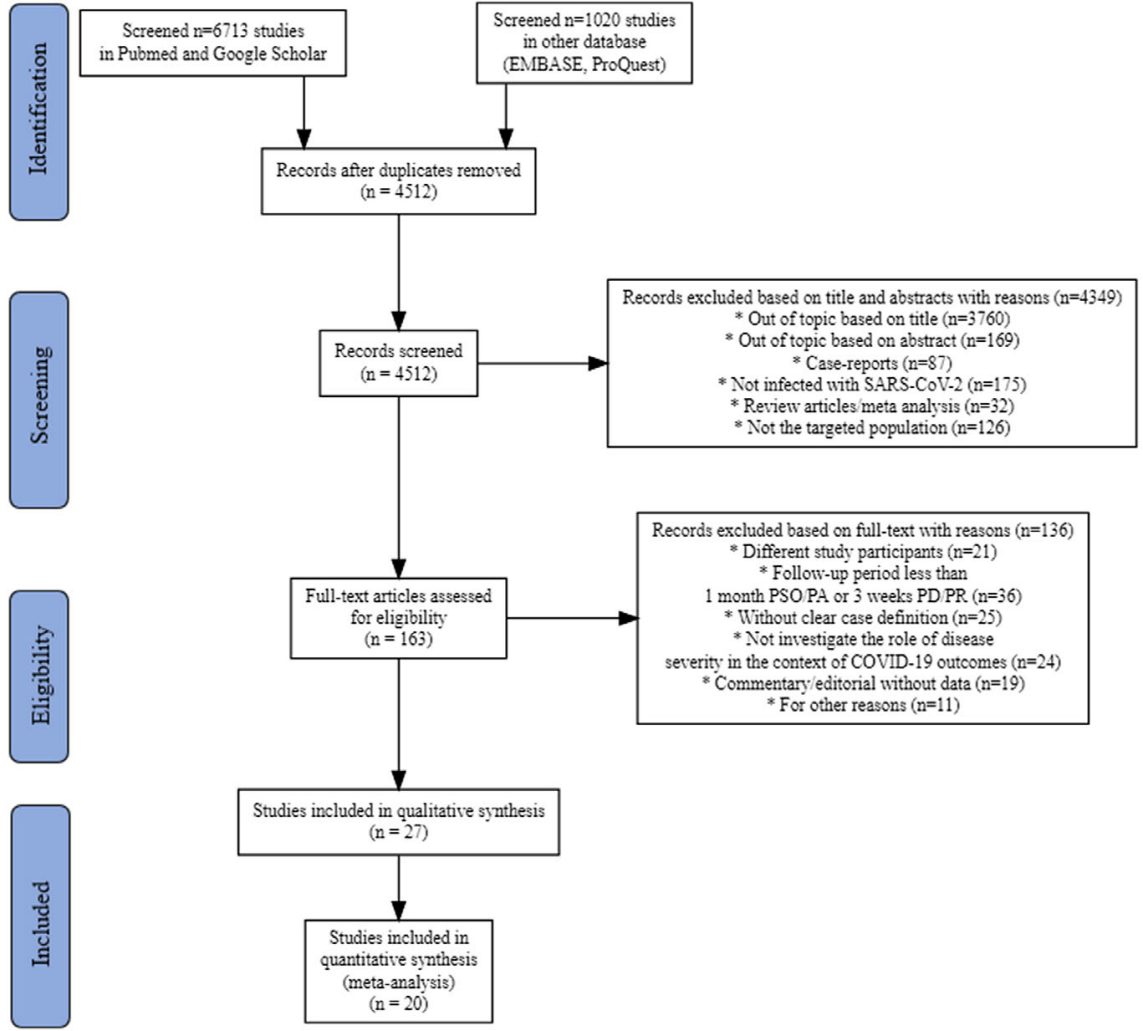
Flow diagram of the literature search

On 30 August 2021, a total of 7733 studies were identified in the initial search of the four databases with the predefined search terms. After removing duplications and evaluating according to elimination and eligibility criteria, a meta-analysis was conducted with 20 studies.(Garrigues *et al.*, 2020; Wang *et al.*, 2020; de Graaf *et al.*, 2021; De Lorenzo *et al.*, 2020; Halpin *et al.*, 2021; Huang *et al.*, 2021a, 2021b; Iqbal *et al.*, 2021; Jacobson *et al.*, 2021; Lerum *et al.*, 2021; Lombardo *et al.*, 2021; Maestre-Muñiz *et al.*, 2021; Meije *et al.*, 2021; Menges *et al.*, 2021; Morin *et al.*, 2021; Qin *et al.*, 2021; Shendy *et al.*, 2021; Sykes *et al.*, 2021; Voruz *et al.*, 2021; Zayet *et al.*, 2021) The details of the study selection process are shown in a PRISMA flow chart (Figure 1).

### Characteristics of studies included and participants

The main characteristics of these 20 studies are shown in Table 1. Most of the studies (*n* = 17, 85%) were single-center cohort and observational studies with a sample size varying from 45 (Voruz *et al.*, 2021) to 1733 (Huang *et al.*, 2021a) patients. Six studies included patients who aged <50 years. The present systematic review and meta-analysis included data from COVID-19 survivors observed between January and December 2020. Most of (70%) the studies evaluated in this meta-analysis included COVID-19 survivors with symptoms persisting beyond 12 weeks of onset of acute infection (long COVID) (Table 2). The follow-up intervals ranged from 23 days to 12 months post-discharge.

**Table 2.** Clinical characteristics of studies on persistent symptoms

Author	Follow Period	Dyspnea		Cough		Fatigue		Anosmia		Chest pain		Palpitation	
	***Initial disease severity (events/severe total, events/nonsevere total)***												
Garrigues *et al.*	>12 week	12/24	38/96	6/24	14/96	14/24	52/96	4/24	9/96	–	–	–	–
Graaf *et al.*	≤12 week	–	–	–	–	–	–	–	–	8/34	7/47	5/34	7/47
Halpin *et al.*	≤12 week	21/32	29/68	–	–	23/32	41/68	–	–	–	–	–	–
Huang *et al.* (a)	>12 week	–	–	–	–	95/117	943/1538	14/117	162/1538	10/117	65/1538	13/117	141/1538
Huang *et al.* (b)	>12 week	–	–	–	–	21/95	234/1185	6/95	51/1185	4/95	88/1185	7/95	110/1185
Iqbal *et al.*	≤12 week	12/13	28/145	9/13	26/145	12/13	119/145	5/13	16/145	12/13	25/145	–	–
Jacobson *et al.*	>12 week	11/22	20/96	0/22	1/96	8/22	28/96	2/22	23/96	2/22	14/96	0/22	7/96
Lerum *et al.*	≤12 week	5/15	32/88	–	–	–	–	–	–	–	–	–	–
Lombardo *et al.*	>12 week	70/189	40/114	–	–	101/189	57/114	49/189	35/114	–	–	–	–
Lorenzo *et al.*	≤12 week	40/126	18/59	–	–	–	–	–	–	–	–	–	–
Maestre *et al.*	>12 week	240/445	104/321	–	–	162/445	110/321	98/445	95/321	29/445	33/321	44/445	24/321
Meije *et al.* (a/b)	≤12 week/			18/115 (a)	28/179 (a)	64/115 (a)	93/179 (a)	14/115 (a)	33/179 (a)	12/115 (a)	18/179 (a)	–	–
	>12 week	13/115 (b)	15/179 (b)	8/115 (b)	9/179 (b)	36/115 (b)	42/179 (b)	9/115 (b)	18/179 (b)	3/115 (b)	5/179 (b)		
Menges *et al.*	>12 week	37/81	59/350	–	–	38/81	195/350	–	–	–	–	–	–
Morin *et al.*	>12 week	25/73	53/405	5/73	16/405	24/73	110/405	6/73	19/405	9/73	25/405	–	–
Qin *et al.*	>12 week	30/248	26/399	18/248	20/399	46/248	41/399	–	–	3/248	3/399	34/248	29/399
Shendy *et al.*	>12 week	–	–	–	–	8/11	44/70	–	–	–	–	–	–
Sykes *et al.*	>12 week	19/27	60/107	5/27	40/107	9/27	44/107	0/27	13/107	1/27	23/107	–	–
Voruz *et al.*	>12 week	–	–	–	–	1/15	5/30	2/15	0/30	–	–	–	–
Wang *et al.*	≤12 week	2/69	0/62	4/69	8/62	0/69	0/62	–	–	0/69	0/62	0/69	0/62
Zayet *et al.*	>12 week	–	–	–	–	–	–	36/121	64/233	–	–	–	–

The table shows the number of each persistent symptom in total severe and total nonsevere cases.

A total of 7840 patients were included. The studies included 1671 (46.8%) females and 4169 (53.2%) males, with mean/median age ranging from 34 (Shendy *et al.*) to 68.8 (Meije *et al.*, 2021) years. The rate of ICU admission was reported in 16 studies as mean 8% (range, 42% (de Graaf *et al.*, 2021) to 2.2% (De Lorenzo *et al.*, 2020)) of reported patients, and four studies did not report this rate (Wang *et al.*, 2020; Maestre-Muñiz *et al.*, 2021; Qin *et al.*, 2021; Shendy *et al.*, 2021). Obesity (BMI ≥ 30) rates were reported in only six studies at mean 22%, while that for CS use was 20.3% according to available data from seven studies. Seven studies (Wang *et al.*, 2020  Huang *et al.*, 2021a, 2021b; Iqbal *et al.*, 2021; Meije *et al.*, 2021; Qin *et al.*, 2021; Voruz *et al.*, 2021) included nonhospitalized patients.

### Pooled ORs of persistent symptoms among COVID-19 survivors

#### Persistent dyspnea

**Figure 2.**
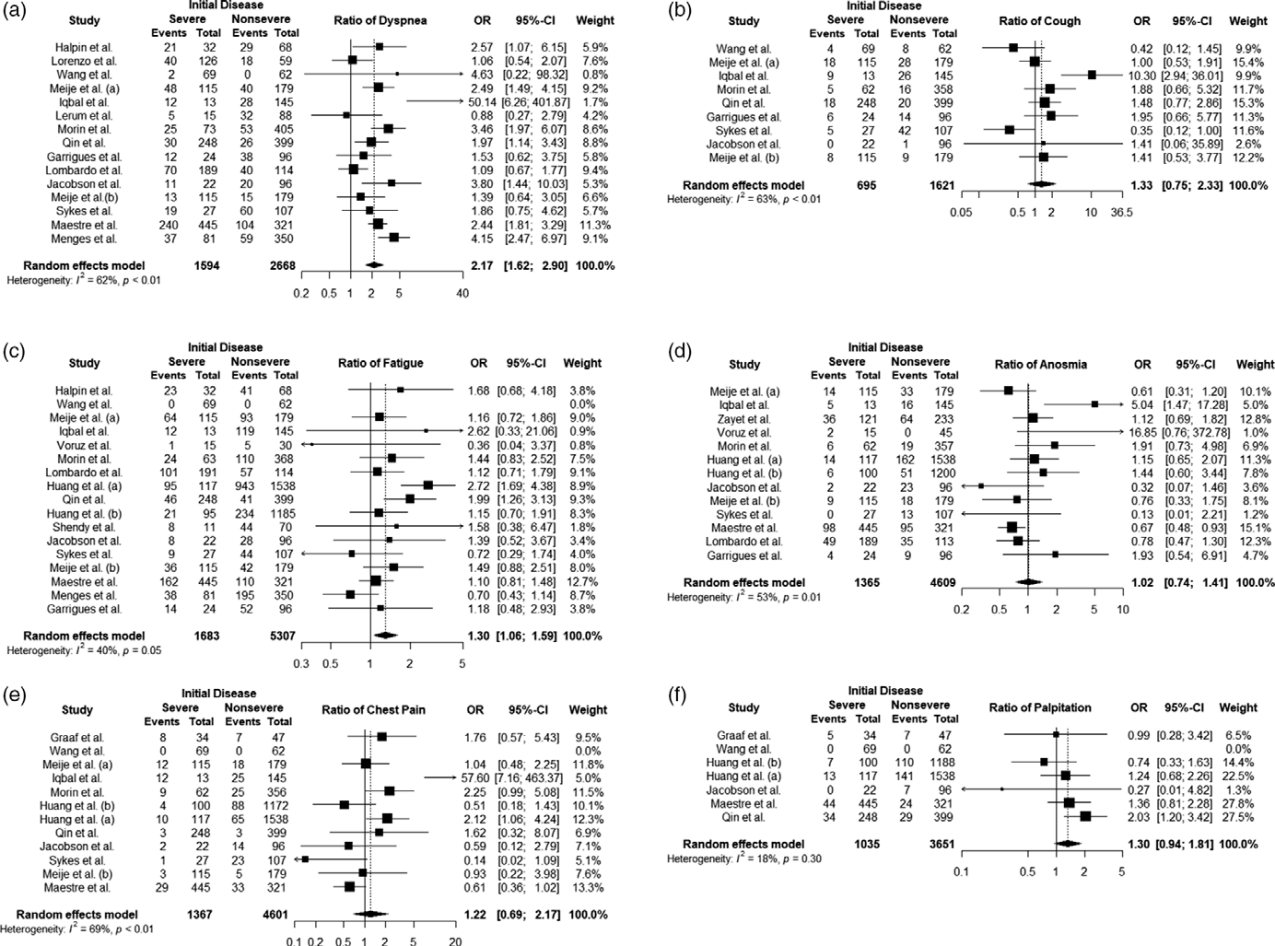
Forest plots of ORs for all persistent symptoms

Fourteen studies including 4270 patients reported persistent dyspnea. Severe survivors of COVID-19 compared to nonsevere had a pooled OR of 2.17 [95%CI 1.62 to 2.90; *P* < 0.001] and a heterogeneity value of *I* ^2^ = 61.6%, Q = 36.48 (*P* = 0.009) (Figure 2a). While 46% of the studies (40.1% weight total) produced a nonsignificant OR for dyspnea, none of the studies did not pose a risk for persistent symptom in nonsevere cases (OR < 1). In addition, in the study of Iqbal *et al.*, although its OR is high (50.14), its effect on pooled OR is 1.7%.

#### Persistent cough

Eight studies including 2382 patients reported persistent cough. Severe survivors of COVID-19 compared to nonsevere had a pooled OR of 1.33 [95%CI 0.75 to 2.33; *P* = 0.325] and heterogeneity value of *I* ^2^ = 62.9%, Q = 21.57 (*P* = 0.005) (Figure 2b). The OR CIs for the persistent symptom of cough in almost all studies (90.1 weight total) included a value of 1. Therefore, the pooled OR was found to be insignificant.

#### Persistent fatigue

Sixteen studies including 7117 patients reported persistent fatigue. Severe survivors of COVID-19 compared to nonsevere had a pooled OR of 1.30 [CI 1.06 to 1.58; *P* = 0.01] and heterogeneity value of *I* ^2^ = 40.5%, Q = 25.19 (*P* = 0.04) (Figure 2c). Five studies (Wang *et al.*, 2020; Iqbal *et al.*, 2021; Jacobson *et al.*, 2021; Menges *et al.*, 2021; Shendy *et al.*, 2021) included patients who aged <50 years.

#### Persistent anosmia

Twelve studies including 6042 patients reported persistent anosmia. Severe survivors of COVID-19 compared to nonsevere had a pooled OR of 1.02 [CI 0.73 to 1.40; *P* = 0.921] and heterogeneity value of *I* ^2^ = 53.3%, Q = 25.70 (*P* = 0.011) (Figure 2d). Although a very high OR (16.85) was obtained from Voruz *et al.*’s study for the persistent symptom of anosmia, the CI of this study includes a value of 1. Only the study of Iqbal *et al.* gives significant OR, its weight is 5%.

#### Persistent chest pain

Eleven studies including 6118 patients reported persistent chest pain. Severe survivors of COVID-19 compared to nonsevere had a pooled OR of 1.22 [CI 0.69 to 2.16; *P* = 0.489] and heterogeneity value of *I* ^2^ = 69.5%, Q = 32.77 (*P* = 0.003) (Figure 2e). For persistent symptom of chest pain, 75% (83% overall weight) of the studies produced an insignificant OR, while the weights of the studies with significant risk consisted of 17.3%.

#### Persistent palpitation

Seven studies including 4752 patients reported persistent palpitation. Severe survivors of COVID-19 compared to nonsevere had a pooled OR of 1.30 [CI 0.93 to 1.81; *P* = 0.112] and heterogeneity value of *I* ^2^ = 18.1%, Q = 6.11 (*P* < 0.295) (Figure 2f). It was concluded that the OR CI formed by each of the studies included in the meta-analysis for palpitation included one value.

### Subgroup analysis

No significant results were found for any persistent symptom when the 12-week time period was accepted as the moderator variable and meta-regression was performed (dyspnea *P* = 0.914, cough *P* = 0.752, fatigue *P* = 0.851, anosmia *P* = 0.591, chest pain *P* = 0.106, and palpitation *P* = 0.684).

### Publication bias and quality analysis

**Figure 3.**
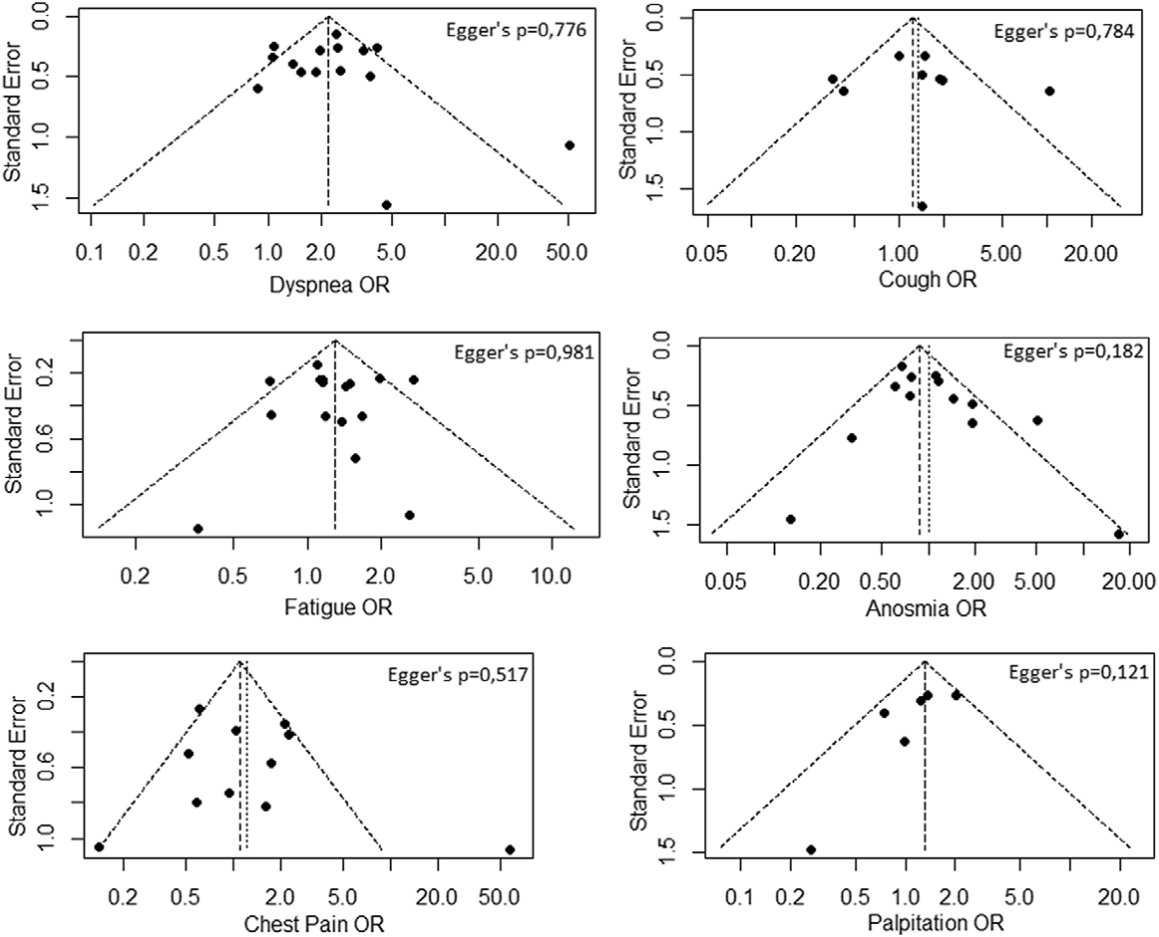
Funnel plots for all persistent symptoms

Figure 3 shows the symmetry of funnel plots and the results of the Egger’s regression analysis, which indicated that no significant publication bias found for any persistent symptom. The average NOS score of the included studies was 5.5 (range, 4–7). There were 3 low-quality studies with a NOS score of ≤4 and 17 high-quality studies with a NOS score of ≥5.

## Discussion

Previous studies evaluating the impact of initial disease severity on the incidence of post-acute and long COVID-19 symptoms have generated inconsistent results (Huang *et al.*, 2021b, 2021a; Kamal *et al.*, 2021). To provide a clearer answer to this question, the current systematic review and meta-analysis aimed to synthesise studies investigating this issue. To the best to our knowledge, the current meta-analysis is the only systematic review to have examined the impact of initial disease severity on the risk of post-acute and long COVID symptoms. The main findings of this meta-analysis were that the risk of cough and chest pain, as well as the risk of anosmia and palpitation, which are common reported respiratory and non-respiratory persistent symptoms, were not affected by the severity of the initial disease. In contrast, compared to patients with less severe acute infection, those who experienced a more severe course were more likely to suffer from persistent dyspnea and fatigue.

The results of this meta-analysis showed that the risk of persistent cough was not affected by the initial disease severity, and this was also valid for chest pain. Although, these findings differ from some previous studies (Song *et al.*, 2021), they are mainly consistent with the current literature (Fernández-de-Las-Peñas *et al.*, 2021). This difference could be due to the subjective nature of self-reporting methods used in these studies. In addition, obtaining a higher risk coefficient for persistent cough and chest pain symptoms in Iqbal’s study compared to other individual studies create heterogeneity. This may be due to the fact that severe cases had a small percentage among all cases in Iqbal’s study, while these patients have experienced relatively higher rates of persistent cough and chest pain symptoms. However, this does not affect the pooled OR much, as these persistent symptoms have a 9.9% versus 5% weight, respectively. Unfortunately, it is rather difficult to interpret these results, since chest pain can be caused by a variety of conditions ranges from myocardial or pulmonary injury to panic attack or musculoskeletal disorders among patients recovering from COVID-19. Furthermore, there are no available data on this issue from previous outbreaks of either SARS-CoV or MERS-CoV infections (Cares-Marambio *et al.*, 2021; Ahmed *et al.*, 2020).

Reports on the risk of persistent palpitation after acute COVID-19 infection regarding the disease severity are highly varied. Qin *et al.* reported a twofold increased risk of having persistent palpitations associated with more severe disease, while others did not find a significant CI for the OR. (Huang *et al.*, 2021b; Jacobson *et al.*, 2021). Broadly consistent with the available literature, the current meta-analysis showed that there was no link between the initial disease severity and the risk of persistent palpitation. This result is in agreement with Puntman’s findings which showed that cardiac involvement in cardiac MR is not influenced by the initial disease severity in individuals who have recovered from acute COVID-19 infection (Puntmann *et al.*, 2020). This systematic review also found no difference in the risk of persistent anosmia between the severity groups. Although the ORs value for anosmia is very high in Voruz *et al.*’s study, the number of patients included in this study is quite low compared to other studies. Therefore, even a low number of patients have experienced persistent anosmia that can cause a higher risk ratio coefficients. However, when individual studies were evaluated according to study’s weight, and since the weight of Voruz *et al.*’s study represents approximately 1%, it was concluded that the pooled OR value for persistent anosmia does not pose a risk. Interestingly, in the study of Maestre *et al.*, moderate–critical disease seemed to be protective against persistent anosmi, and this finding is supported by the results previously reported by Lechien *et al.* of more persistent anosmia in mild COVID-19 patients than in moderate-to-critical patients. These results were attributed to a better local immunological response in mild patients that limited the virus spread but could result in a stronger impairment of olfactory cells (Lechien *et al.*, 2021).

The current meta-analysis showed that individuals with critical–severe initial disease were more than twice as likely to suffer from persistent dyspnea compared with those had less severe initial disease. Therefore, it seems important that patients with severe/critical initial disease should be followed more closely than patients with moderate/mild initial disease, and these patients should be referred for pulmonary rehabilitation. Another important finding was that although a reduction in the OR was seen over time, severe–critical patients still had a 4.15 and 2.44-fold higher risk of persistent dsypnea compared to less severe patients even beyond 6 and 12 months, respectively (Maestre-Muñiz *et al.*, 2021; Menges *et al.*, 2021). These results are consistent with those of previous studies which have reported prolonged lung diffusion impairment and abnormal findings in the chest CT at 6 and 12 months in critically ill patients (Huang *et al.*, 2021b; Nalbandian *et al.*, 2021). It seems possible that these results are due to prolonged inflammation in critical-severe patients which was not seen in nonsevere patients (Del Valle *et al.*, 2020; Maltezou *et al.*, 2021). This hypothesis is supported by the results of Ortelli *et al.* showing that patients with post-COVID manifestation had markedly elevated C-reactive protein (CRP) and IL-6 during the acute phase of COVID-19 infection (Ortelli *et al.*, 2021). In summary, the findings reported here suggest that critical–severe patients have a much longer recovery time following acute illness with higher odds of persistent dyspnea than those with less severe acute illness.

It is well known that critical illness with prolonged stay in ICU or hospital is associated with a loss of muscle mass and a decreased lung capacity (Saccheri *et al.*, 2020; Vanhorebeek *et al.*, 2020). Therefore, as expected, a significantly higher likelihood of experiencing persistent fatigue was determined after a more severe course of initial disease compared with a less severe course. However, there are conflicting reports on the role of initial disease severity in the risk of persistent fatigue. While some study results (Huang *et al.*, 2021a; Qin *et al.*, 2021) support the current study findings, others have failed to find a link between the risk of persistent fatigue and initial disease severity (Menges *et al.*, 2021; Sykes *et al.*, 2021). This inconsistency may be due to the variance in patient characteristics such as corticosteroid use during acute infection, which was reported only by Huang *et al.* as significantly higher in critically ill patients, while there was no differences between two groups regarding age, gender, and the presence of chronic lung disease (Huang *et al.*, 2021a).

### Limitations

A major limitation of the present systematic review and meta-analysis was the substantial heterogeneity of the summarized studies in respect of mean ages, gender, the rate of ICU admission, CS use, and follow-up periods. Therefore, the random effects model was used to pool the data. This study was also limited by the absence of randomized studies on this topic, and that all of the reviewed studies were observational in nature. Another limitation of the present study is that, in some included studies, it is not clear whether cases that we have classified as nonsevere include cases with mild initial disease severity, and whether cases that we have classified as severe include cases with critical initial disease severity.

## Conclusion

The results of this systematic review and meta-analysis revealed that the risks of persistent cough, chest pain, anosmia, and palpitation were not affected by the initial disease severity. The research has also concluded that the risk of persistent dyspnea and fatigue were significantly higher in COVID-19 survivors with a severe initial disease than in those with a nonsevere initial disease. Moreover, this meta-analysis showed that being in the post-acute or long COVID phase did not affect the risk of symptoms. Clinicians should be alert to potential persistent cough and chest pain as well as anosmia and palpitation in all COVID-19 survivors, which are not limited to patients with critical–severe initial disease.

## Data Availability

Data available within the article or its supplementary materials.

## PROSPERO registration number

CRD42021272990.
